# Ventricular Anatomy Across CT and MRI in Hydrocephalus: A Retrospective Study

**DOI:** 10.3390/diagnostics16030491

**Published:** 2026-02-05

**Authors:** Andrada-Iasmina Roşu, Laura Andreea Ghenciu, Dan Cristian Roşu, Emil-Radu Iacob, Emil Robert Stoicescu, Roxana Stoicescu, Alexandra Ioana Dănilă, Sorin Lucian Bolintineanu

**Affiliations:** 1 Doctoral School, University of Medicine and Pharmacy “Victor Babes”, Eftimie Murgu Square No. 2, 300041 Timisoara, Romania; iasmina.rosu@umft.ro; 2 Department of Anesthesia and Intensive Care, University of Medicine and Pharmacy “Victor Babes”, Eftimie Murgu Square No. 2, 300041 Timisoara, Romania; 3 Department of Functional Sciences, Discipline of Pathophysiology, University of Medicine and Pharmacy “Victor Babes”, 300041 Timisoara, Romania; bolintineanu.laura@umft.ro; 4 Center for Translational Research and Systems Medicine, University of Medicine and Pharmacy “Victor Babes”, 300041 Timisoara, Romania; 5 1st Surgery Clinic, University of Medicine and Pharmacy “Victor Babes”, Eftimie Murgu Square No. 2, 300041 Timisoara, Romania; 6 Department of Pediatric Surgery, University of Medicine and Pharmacy “Victor Babes”, 300041 Timisoara, Romania; 7 Department of Radiology and Medical Imaging, University of Medicine and Pharmacy “Victor Babes”, Eftimie Murgu Square No. 2, 300041 Timisoara, Romania; stoicescu.emil@umft.ro; 8 Research Center for Pharmaco-Toxicological Evaluations, University of Medicine and Pharmacy “Victor Babes”, Eftimie Murgu Square No. 2, 300041 Timisoara, Romania; 9 Field of Applied Engineering Sciences, Specialization Statistical Methods and Techniques in Health and Clinical Research, Faculty of Mechanics, Politehnica University Timisoara, Mihai Viteazul Boulevard No. 1, 300222 Timisoara, Romania; 10 Department of Anatomy and Embriology, University of Medicine and Pharmacy “Victor Babes”, Eftimie Murgu Square No. 2, 300041 Timisoara, Romania; roxana.iacob@umft.ro (R.S.); alexandra.danila@umft.ro (A.I.D.); s.bolintineanu@umft.ro (S.L.B.)

**Keywords:** hydrocephalus, Evans index, ventriculomegaly, computed tomography, magnetic resonance imaging, ventriculoperitoneal shunt

## Abstract

**Background/Objectives**: Hydrocephalus is a complex neurological disorder marked by abnormal cerebrospinal fluid dynamics and ventricular enlargement. Despite breakthroughs in neuroimaging, diagnosis and longitudinal the application of imaging markers for the diagnosis and longitudinal monitoring of hydrocephalus remains challenging in routine clinical practice. The present study examines the behavior and cross-modality agreement of commonly used linear ventricular measurements under routine imaging conditions, at a single Romanian tertiary-care center characterized by heterogeneous acquisition protocols and limited availability of advanced volumetric techniques. **Methods**: We conducted a single-center retrospective observational study of 68 adults with hydrocephalus. Linear ventricular metrics, including Evans index and third-ventricle width, were measured on all available CT and MRI scans. CT–MRI agreement was assessed using paired examinations within a 90-day window. Longitudinal changes were analyzed using first–last and pre–post VP shunt comparisons. Associations between baseline imaging features and VP shunt placement were evaluated using rule-based and odds ratio analyses. **Results**: CT and MRI measurements demonstrated strong agreement for both Evans index (r = 0.93) and third-ventricle width (r = 0.90), with minimal systematic bias. Longitudinal analyses demonstrated small-magnitude changes in ventricular size following intervention, with substantial inter-individual variability. VP utilization increased across Evans index strata, reaching 100% in patients with values ≥0.50. Transependymal cerebrospinal fluid exudation showed the strongest association with subsequent VP shunting. Imaging-based rules exhibited expected trade-offs between sensitivity and specificity. **Conclusions:** Standard linear ventricular parameters exhibited adequate cross-modality agreement and clinically important longitudinal behavior in this cohort. While insufficient as standalone predictors, these readily available imaging markers remain important tools when combined with a comprehensive clinical assessment.

## 1. Introduction

Hydrocephalus constitutes a prevalent but extremely diverse neurological disorder defined by abnormal cerebrospinal fluid (CSF) dynamics and ventricular enlargement [1,2]. It encompasses a heterogeneous group of disorders with distinct pathophysiological mechanisms, ranging from acute obstruction and inflammatory responses in post-hemorrhagic hydrocephalus to chronic disturbances of CSF dynamics in idiopathic normal-pressure hydrocephalus, often coexisting with neurodegenerative pathology [1,2]. Despite significant breakthroughs in neuroimaging and neurosurgical procedures, hydrocephalus diagnosis, monitoring, and treatment decisions remain difficult in everyday clinical practice [3]. This issue stems from a wide range of etiologies, diverse clinical presentations, and a lack of widely accepted imaging biomarkers that accurately represent disease severity, progression, and therapy responsiveness [1,4,5].

Neuroimaging is essential in the evaluation of hydrocephalus, providing a foundation for diagnosis, etiologic assessment, longitudinal monitoring, and post-treatment evaluation [6,7]. The most often employed modalities are computed tomography (CT) and magnetic resonance imaging (MRI), with each providing complementary benefits [8,9]. In real-world clinical settings, imaging methods are rarely standardized, and patients frequently receive numerous examinations across different modalities and time points based on clinical urgency, accessibility, and institutional policy [10,11].

Simple linear ventricular measurements have long been used as practical markers of ventricular enlargement due to their simplicity and reproducibility. However, the commonly cited thresholds for these measures were derived primarily from Western European and North American populations and should not be interpreted as biologically universal or population-independent norms. Their clinical utility lies less in absolute cut-off values and more in their role as operational reference points that facilitate communication, longitudinal comparison, and decision support within specific clinical contexts [12]. The Evans index, which is defined as the ratio of maximum frontal horn width to maximum inner skull diameter, has long been utilized as a screening tool for ventriculomegaly [13,14]. Additional linear measures, such as third-ventricle width, temporal horn enlargement, frontal and occipital horn ratio and callosal angle, are frequently reported and aid in the radiologic assessment of hydrocephalus severity and subtype [9,15,16]. Despite their extensive use, these measures are not without limitations. They only provide a rough estimate of ventricular size, are affected by age-related brain atrophy and skull morphology [17,18]. Furthermore, their ability to identify minor longitudinal changes has been questioned [19]. Baseline ventricular anatomy also exhibits substantial inter-individual variability and is influenced by age-related brain changes, comorbidity burden, and broader population-specific health trajectories.

Variability caused by different acquisition techniques and cross-modality assessment is a persistent challenge in hydrocephalus imaging [20,21]. While MRI is frequently regarded as the gold standard for ventricular and parenchymal examination, CT remains the preferred modality in many clinical settings [22,23]. As a result, basic linear measurements remain the most readily available and routinely used imaging techniques worldwide, especially in settings where CT is the primary modality and MRI access is limited [12]. Longitudinal imaging assessment is equally challenging due to heterogeneous follow-up intervals, mixed imaging modalities across time points, and clinically driven rather than protocolized imaging schedules [24]. Patients with hydrocephalus frequently receive repeated imaging to track disease progression, guide treatment decisions, and assess post-intervention results. However, follow-up intervals, imaging modalities, and measurement availability are extremely uneven [22].

Recent advances in artificial intelligence and automated image processing have yielded promising volumetric and prognostic approaches to hydrocephalus diagnosis [25,26,27]. However, these methods frequently necessitate specialized infrastructure, curated datasets, and extensive validation, which limits their immediate use in many institutions [28,29,30]. Against this background, there is a clear need for research on the performance, agreement, and longitudinal behavior of basic ventricular imaging markers.

The present study addresses this gap by examining a retrospective cohort of adults with hydrocephalus evaluated under routine clinical conditions at a single tertiary center. Linear ventricular measurements are treated as geometric descriptors of ventricular size rather than as population-normalized diagnostic constructs. We aimed to assess cross-modality agreement between CT- and MRI-derived linear ventricular measurements, characterize longitudinal changes in ventricular size over time and following CSF diversion, and evaluate the relationship between baseline imaging features and subsequent VP shunt placement. Through this approach, we aim to contribute evidence that bridges the gap between advanced imaging research and everyday clinical practice, reinforcing the role of accessible, reproducible imaging metrics while acknowledging their limitations.

## 2. Materials and Methods

### 2.1. Study Design

We performed a single-center, retrospective observational studyat the Timis County Emergency Hospital ‘Pius Brânzeu’ Timișoara, Romania, including consecutive patients evaluated between January 2023 and December 2024 with a clinical and radiological diagnosis of hydrocephalus. This diagnosis of hydrocephalus was established by the treating neurology or neurosurgery team, supported by radiological evidence of ventricular enlargement and CSF circulation disturbance. All analyses were conducted on de-identified records according to institutional policy and in line with STROBE recommendations for observational studies.

### 2.2. Participants

Eligible patients were identified using consecutive sampling from institutional clinical and radiological records. All adult patients evaluated for suspected or confirmed hydrocephalus at the Clinical County Emergency Hospital ‘Pius Brânzeu’ Timișoara during the study period were screened for eligibility. Inclusion criteria were: (i) age ≥ 18 years; (ii) evaluation between January 2023 and December 2024; (iii) a clinical diagnosis of hydrocephalus established by the treating neurology or neurosurgery team; (iv) radiological evidence of ventricular enlargement and CSF circulation disturbance. Patients were included irrespective of hydrocephalus etiology or management strategy; inclusion was not limited to cases undergoing VP shunt placement.

At least one cranial CT or MRI examination of sufficient quality was required for review. Patients were excluded if imaging quality was insufficient for reliable linear ventricular measurements, including cases with severe motion artifacts, incomplete ventricular coverage, or postoperative changes obscuring relevant anatomic landmarks. During the study period, 96 patients were identified as having a clinical diagnosis of hydrocephalus. Of these, 28 were excluded, resulting in a final analytic cohort of 68 patients. Because imaging availability and completeness varied across individuals and time points, analytic denominators differed by variable and analysis; these are explicitly reported throughout. A complete-case approach was used for all analyses, and no imputation of missing values was performed.

### 2.3. Clinical Data and Variables

Demographic variables included age, sex and race/ethnicity at the time of the first available imaging examination. All patients were treated within a publicly funded healthcare system providing universal access. Etiology of hydrocephalus was abstracted from clinical documentation and categorized into predefined groups: post-hemorrhagic, tumor-related, post-infectious, idiopathic/normal-pressure hydrocephalus, other secondary causes, or unknown. Device-related variables included placement of an external ventricular drain (EVD) and VP shunt, recorded as binary indicators of exposure at any point during follow-up (“ever-EVD” and “ever-VP”). Dates of device placement were recorded when available to allow temporal pairing with imaging studies.

### 2.4. Imaging Acquisition and Selection

All available cranial CT and MRI examinations performed during the study period were screened. Imaging protocols were not standardized and reflected routine clinical indications, including emergency assessment, inpatient monitoring, and outpatient follow-up, for both CT and MRI examinations. When multiple examinations were available for a given patient, scan dates were used to establish temporal sequences and to enable longitudinal and cross-modality analyses, with examinations systematically ordered in ascending chronological order for each patient. For analyses requiring modality pairing, CT–MRI pairs were identified based on temporal proximity. Imaging acquisition and follow-up reflected institutional practice patterns at our center and were not intended to represent standardized or universally adopted imaging protocols.

### 2.5. Imaging Measurements

CT examinations were performed using multi-detector scanners (64–128 slice) operating under routine clinical protocols, including non-contrast axial acquisitions with slice thickness ranging from 0.6 to 5 mm. MRI studies were acquired on 1.5T and 3T scanners using standard clinical sequences, including axial T1-weighted and T2-weighted images with slice thicknesses of 3–5 mm. The Evans index was defined as the ratio of the maximal frontal horn width of the lateral ventricles to the maximal inner skull diameter on the same axial slice [14]. Third-ventricle (V3) width was measured as the maximal transverse diameter on axial images at or near the level of the foramina of Monro [31]. The callosal angle was measured on coronal images reconstructed perpendicular to the anterior–posterior commissural plane at the level of the posterior commissure, defined as the angle between the medial walls of the lateral ventricles [32]. Temporal horn width was measured as the maximal transverse diameter on axial images [33]. Measurements were performed using digital caliper tools available on the institutional PACS system. Etiologic categories were recorded for descriptive purposes. Linear ventricular measurements were analyzed as continuous variables or used for descriptive stratification only. No diagnostic classification or validation of population-independent cutoff values was performed or intended.

Transependymal CSF exudation was recorded as present or absent based on periventricular hypoattenuation on CT or periventricular hyperintensity on T2-weighted or fluid-attenuated inversion recovery (FLAIR) MRI sequences [34]. Ventricular configuration was categorized as bi-, tri-, or tetraventricular enlargement according to radiology reports and imaging review. Certain imaging features were not assessable in all patients due to variability in imaging quality and protocols. For the features that could not be reliably assessed in all patients, results are reported as *n*/*N*, where *n* reflects the number of evaluable examinations.

All measurements were performed by trained clinicians with experience in neuroimaging interpretation (radiology and neurosurgery backgrounds). Measurements were conducted independently for CT and MRI examinations. In cases of ambiguity or borderline measurements, images were reviewed jointly to reach consensus. Formal inter-operator variability was not quantified. Operators were not involved in clinical decision-making regarding VP shunt placement at the time of measurement. To minimize measurement bias, operators adhered to predefined anatomical landmarks and measurement conventions, and ambiguous cases were reviewed jointly to achieve consensus. Measurements were performed without access to longitudinal outcomes or treatment decisions when feasible.

To assess intra-observer reliability, a randomly selected subset of 20 imaging examinations was re-measured by the same observer after a washout period of at least two weeks, blinded to the initial measurements. Intra-observer agreement for linear ventricular measurements was evaluated using intraclass correlation coefficients (ICC; two-way mixed-effects model, absolute agreement) and Bland–Altman analysis.

### 2.6. Outcomes

The primary outcome was VP shunt placement at any time during follow-up (“ever-VP”), selected as a clinically meaningful endpoint reflecting the decision to initiate long-term CSF diversion. Secondary outcomes included ever-EVD placement and the operating characteristics of simple imaging-based rules derived from ventricular measurements, evaluated against subsequent VP shunt placement. VP shunt placement was analyzed as a contextual clinical outcome reflecting integrated, individualized decision-making rather than as an independent validation standard for imaging measurements

### 2.7. Longitudinal Assessments

Two longitudinal analyses were prespecified. First, the last-to-first change was calculated for patients with two or more dated imaging examinations by subtracting the initial measurement from the final available measurement, irrespective of imaging modality. This approach was chosen to capture overall directional change in ventricular size during follow-up under real-world imaging conditions within a tertiary-care hospital.

Second, in patients who underwent VP shunt placement, pre–post change was assessed by comparing the last available pre-operative measurement with the first post-operative measurement. The interval between surgery and the post-operative scan was recorded to contextualize early imaging changes following shunting.

### 2.8. CT–MRI Pairing and Agreement

To assess cross-modality agreement, patients with both CT and MRI examinations were matched by identifying the closest CT–MRI pair within a 90-day window, minimizing the absolute time difference between examinations. If multiple pairs met this criterion, the earliest pair was selected. Agreement between modalities was evaluated using Pearson correlation coefficients, Bland–Altman analysis with mean bias (MRI minus CT) and 95% limits of agreement, and the median absolute difference as a robust measure of dispersion.

### 2.9. Statistical Analysis

Continuous variables are reported as medians with interquartile ranges due to non-normal distributions and bounded measurement scales. Categorical variables are summarized as counts and percentages. Associations between baseline binary imaging features and VP shunt placement were evaluated using Fisher’s exact test, with odds ratios and exact *p*-values reported. Operating characteristics of prespecified imaging-based rules were calculated as sensitivity, specificity, positive predictive value, negative predictive value, and balanced accuracy. All tests were two-sided with a significance level of *p* < 0.05. No formal adjustment for multiple comparisons was applied, given the exploratory nature of the association analyses. Pearson correlation coefficients were used to assess the strength of linear association between CT and MRI measurements at the group level. Agreement between modalities was further evaluated using Bland–Altman analysis to quantify systematic bias and limits of agreement.

Longitudinal imaging data were incomplete, with quantifiable Evans index change available in only a subset of patients. No assumptions were made regarding the randomness of missing data, and formal missing-data modeling or imputation was not performed. Longitudinal analyses were therefore treated as exploratory, and results were interpreted cautiously in light of potential systematic missingness related to clinical follow-up patterns and care pathways. For the association analyses, *p*-values were adjusted for multiple comparisons using the Holm–Bonferroni method (family-wise error rate 0.05).

### 2.10. Ethics

This study was approved by the Ethics Committee of Timis County Emergency Clinical Hospital (approval number 574/16 October 2025). The analysis was conducted on de-identified data, involved no interventions, and posed no additional risk to patients.

## 3. Results

### 3.1. Baseline Characteristics

**Figure 1:**
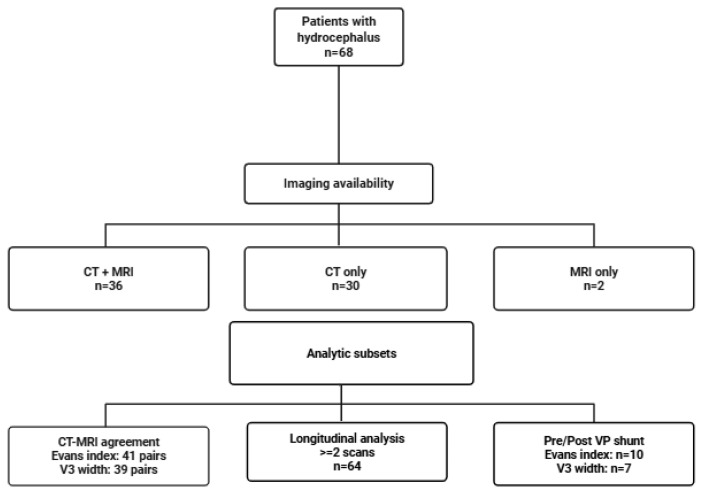
Study flow and analytic subsets derived from the single-center adult hydrocephalus cohort. The diagram illustrates patient identification and the resulting analytic subsets used for cross-modality (CT–MRI) and longitudinal analyses.

**Figure 2:**
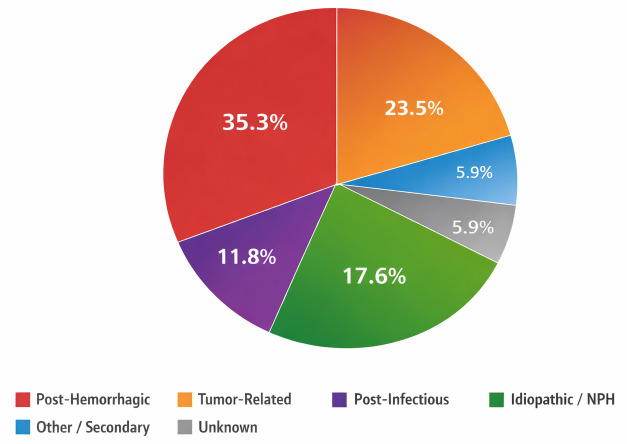
Distribution of hydrocephalus etiologies in the study cohort (*N* = 68). The pie chart illustrates the proportional distribution of etiologic categories expressed as percentages.

We analyzed 68 patients with hydrocephalus; overall, 53% of patients were women, and the median age of the cohort was 61 years. Imaging availability was high, with just over half having both CT and MRI (52.9%), most of the remainder had CT alone (44.1%), and a small minority had MRI alone (2.9%) (Figure 1). Baseline imaging characteristics were consistent with a cohort enriched for clinically relevant ventriculomegaly (Table 1). Transependymal CSF exudation was documented in nearly three-fifths of reports with explicit descriptors. Ventricular configuration was predominantly triventricular, followed by tetraventricular patterns. Reported etiologies were chiefly post-hemorrhagic and tumor-related, with smaller post-infectious and idiopathic/ normal pressure hydrocephalus (NPH) subsets (Figure 2). Callosal angle narrowing and temporal horn enlargement were common, supporting the presence of advanced ventricular dilation across etiologic subgroups. Intra-observer reliability was high for linear ventricular measurements. The intraclass correlation coefficient was 0.92 for Evans index and 0.89 for third-ventricle width, indicating good to excellent agreement. Bland–Altman analysis demonstrated minimal systematic bias with narrow limits of agreement.

### 3.2. Device Utilization

During follow-up, cerebrospinal fluid diversion devices were frequently used, with VP shunting slightly more common than external ventricular drain (EVD) placement (Table 2). Several patients underwent more than one form of CSF diversion during their clinical course. Overall device utilization in this group suggests significant neurosurgical intervention, which is consistent with the considerable ventricular enlargement and imaging severity reported at baseline.

### 3.3. Longitudinal Change

Of the 68 patients included, 64 (94.1%) had at least 2 dated scans, with a median first–last interval of 33 days. Quantitative last–first change was available for Evans index in 22/68 (32.4%) and for third-ventricle width in 16/68 (23.5%). Over the observed follow-up interval, ventricular size demonstrated a small net reduction in most patients, with considerable inter-individual variability in both direction and magnitude of change, reflecting heterogeneity in underlying pathophysiology, disease stage, and clinical intervention. (Table 3). The Evans index showed a median change of −0.02. The third-ventricle width changed by −1.6 mm. The negative medians, together with upper interquartile range (IQR) bounds near zero, indicate small net reductions over the observed intervals, with some cases showing little change or slight increases. Among shunted patients, paired pre– and post–measurements were available in 38.5% of cases for Evans index and 26.9% of cases for third-ventricle width. The Evans index decreased by −0.04 and the third-ventricle width by −3.0 mm from the last pre-operative to the first post-operative scan. Post-operative imaging occurred 7 days after VP.

Marked inter-individual variability was observed in ventricular response following VP shunt placement, ranging from substantial reduction to minimal or no measurable change, indicating heterogeneous response patterns within the cohort.

### 3.4. CT-MRI Agreement

**Figure 3:**
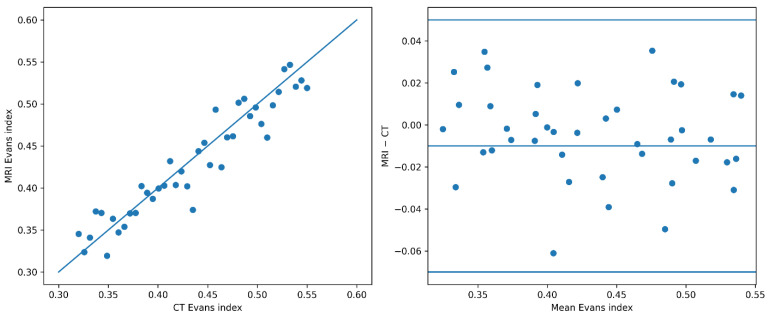
Concordance of Evans index measurements across imaging modalities.

Using nearest examinations within a 90-day window, we identified 41 paired CT–MRI examinations for Evans index assessment and 39 paired examinations for third-ventricle width; remaining patients did not undergo both imaging modalities within this interval. Across these paired examinations, CT- and MRI-derived measurements demonstrated high concordance, with minimal systematic bias and relatively narrow limits of agreement (Table 4, Figure 3). However, CT–MRI agreement analyses were based on a relatively small subset of patients, which limits the precision of agreement estimates and warrants cautious interpretation.

For the Evans index, agreement was particularly strong (r = 0.93), with a small negative mean bias (−0.01) and narrow 95% limits of agreement (−0.07 to +0.05), indicating minimal systematic underestimation on MRI relative to CT. The median absolute difference between modalities was low (0.02). Similarly, third-ventricle width showed a strong correlation between CT and MRI (r = 0.90), with a modest positive bias (+0.6 mm). The Bland–Altman analysis demonstrated limits of agreement for third-ventricle width ranging from −3.2 to +4.4 mm, reflecting the observed measurement variability between CT and MRI in this cohort. The median absolute difference for third-ventricle width was 1.3 mm.

These agreement metrics describe cohort-level measurement consistency across imaging modalities and do not imply equivalence of clinical meaning or decision-making at the individual patient level.

(A) Scatter plot showing the relationship between CT- and MRI-derived Evans index measurements across paired examinations, with the dashed line indicating identity.
(B) Bland–Altman analysis of Evans index measurements obtained from CT and MRI in adult patients with hydrocephalus. The plot displays the mean difference between modalities (solid line) and the 95% limits of agreement (dashed lines). Each point represents a paired CT–MRI examination from the same patient.

### 3.5. Binary Radiologic Markers and VP Shunting

Across available records (*n* varying by imaging feature), transependymal cerebrospinal fluid (CSF) exudation was associated with the largest observed effect size for subsequent ventriculoperitoneal (VP) shunt placement (odds ratio [OR] 3.33); however, this association did not reach statistical significance. (Table 5). In contrast, other imaging features showed weaker associations with VP utilization. Among the evaluated binary imaging markers, none demonstrated a statistically significant association with VP shunt placement. The presence of intracranial hemorrhage (OR 0.80), mass effect (OR 0.67), and cisternal effacement (OR 1.33) was not significantly associated with subsequent VP shunt placement. These associations describe alignment with observed clinical decision-making rather than serving as independent biological markers of disease severity. After Holm–Bonferroni adjustment for multiple comparisons, none of the baseline imaging markers showed a statistically significant association with VP shunt placement.

### 3.6. VP Utilization by Evans-Index Strata

As shown in Table 6, VP shunt utilization increased progressively across Evans index strata, calculated as the proportion of patients within each stratum who underwent VP shunt placement. Evans index strata are presented descriptively across the mixed-etiology cohort and do not imply equivalent biological meaning or treatment thresholds across hydrocephalus subtypes. No patients with an Evans index of 0.30–0.39 underwent VP shunt placement, whereas VP use was observed in half of the patients with Evans index values of 0.40–0.49. In contrast, all patients with Evans index values ≥0.50 received VP shunts.

### 3.7. Rule-Based Prediction of Ever-VP

Simple imaging-based rules showed trade-offs between sensitivity and specificity when evaluated against subsequent VP shunt placement (Table 7). More restrictive criteria increased specificity at the expense of sensitivity, whereas lower thresholds favored sensitivity with reduced specificity.

Across the patients, Evans ≥ 0.40 balanced sensitivity and specificity (66.7% and 65.0%). Adding transependymal edema increased specificity to 85.0% with lower sensitivity (46.7%). A more permissive threshold (Evans ≥ 0.35) yielded higher sensitivity (80.0%) but lower specificity (45.0%). Transependymal edema alone showed high specificity (96.7%) with low sensitivity (33.3%).

## 4. Discussion

Within routine clinical imaging practice, linear ventricular measures demonstrated cross-modality agreement and directionally consistent changes following CSF diversion. Imaging-based criteria were associated with subsequent VP shunt use, supporting the practical utility of commonly applied ventricular markers in hydrocephalus assessment. Rather than relying on standardized imaging protocols or controlled follow-up, this study reflects the range of clinical and imaging scenarios encountered in routine hydrocephalus evaluation at a single Romanian tertiary-care hospital.

Linear indices such as Evans’ index provide only a coarse approximation of ventricular enlargement. Other studies have explored automated CT-based volumetric measures derived using deep learning, including transfer-learning pipelines that leverage MRI-derived labels and manual CT annotations in idiopathic normal pressure hydrocephalus (iNPH) cohorts [35,36]. Previous research has shown that these metrics maintain good cross-modality agreement and clinical relevance. Recent work comparing CT, conventional MRI, and mobile low-field MRI reported excellent agreement for Evans index and ventricular width measurements, despite heterogeneous acquisition protocols and real-world follow-up conditions providing conceptual consistency regarding measurement behavior across modalities [37]. In a cohort of definite iNPH patients, Takagi et al. reported only moderate interobserver agreement for Evans’ index measurements and demonstrated that more than 30% of patients would be missed when relying solely on this threshold [21].

The study of Kockum showed generally substantial to almost perfect agreement between readers and imaging modalities for most iNPH radiological features, such as Evans’ index, temporal horn size, Sylvian fissures, and callosal angle, with lower agreement for white matter changes and focally enlarged sulci [38]. The consistency observed between CT- and MRI-derived Evans index and third-ventricle width supports the interchangeability of these modalities for linear ventricular assessment. Previous comparative studies have reported similar findings under controlled conditions [8,37]; the results extend this evidence to temporally separated examinations obtained during routine care in our institution, reinforcing the clinical utility of CT-based measurements when MRI is unavailable or delayed.

Ventricular size reduction following VP shunt placement is a commonly expected radiologic finding, although the magnitude and timing of change vary considerably across patients and etiologies [39]. Prior research has shown that clinical improvement may precede or occur independently of substantial radiologic normalization, particularly in adult and normal-pressure hydrocephalus [39,40,41]. Longitudinal MRI studies in iNPH have shown that postoperative changes may manifest predominantly as redistribution of CSF spaces and changes in indices such as callosal angle or z-axial measures, while the Evans index may change little over time, highlighting its limited sensitivity for tracking shunt-related morphologic recovery in some cohorts [41]. In our cohort, ventricular measurements demonstrated modest but consistent reductions on early post-operative imaging, supporting the notion that large immediate changes should not be expected in all patients. These data emphasize the need to interpret post-shunt imaging in conjunction with clinical state, rather than as a standalone indicator of therapeutic efficacy. Longitudinal analyses demonstrated only small absolute changes in linear ventricular measurements over time, and the clinical significance of these modest variations remains uncertain. Given the limited magnitude of observed changes, it is difficult to determine whether such differences reflect true disease progression or therapeutic response, or instead fall within the range of expected measurement variability, particularly under non-standardized imaging conditions.

Transependymal CSF exudation is a well-described marker of elevated intraventricular pressure and acute hydrocephalus [6,34]. While its presence has been associated with disease severity, it is not considered independently diagnostic [34,42]. In this study, transependymal exudation showed the largest effect size among binary imaging markers associated with subsequent VP shunting, albeit without statistical significance. This pattern suggests that transependymal edema may increase specificity when interpreted alongside ventricular size indices, supporting its role as a complementary rather than standalone feature in clinical decision-making.

VP shunt placement reflects an integrated clinical decision that incorporates imaging findings, neurological status, symptom progression, and underlying etiology [43,44,45]. As such, imaging-based rules evaluated against VP utilization do not represent the prediction of a biological endpoint but rather alignment with clinical practice [44,45]. The observed trade-offs between sensitivity and specificity across different Evans index thresholds reflect this reality, highlighting that ventricular measures should accompany, rather than replace, full clinical assessment. This perspective aligns with broader literature cautioning against overreliance on single imaging metrics or algorithmic thresholds [29]. The inherent difficulty of predicting shunt responsiveness in iNPH has also been demonstrated in large registry-based studies using advanced multimodal statistical models; notably, a Disease State Index approach integrating clinical, imaging, CSF, and genetic data achieved only modest discriminatory performance between shunt responders and non-responders, underscoring the limitations of algorithmic prediction in this context [45]. The heterogeneity of ventricular response following VP shunt placement highlights an important and unresolved challenge in hydrocephalus management. Substantial reductions in ventricular size in some patients contrasted with minimal morphologic change in others, despite similar interventions. This variability reflects a combination of etiologic differences, disease chronicity, baseline ventricular compliance, timing of shunt placement, and coexisting neurodegenerative or vascular pathology.

The use of a complete-case analytic approach resulted in variable denominators across comparisons and may have introduced selection bias. Patients with paired CT and MRI examinations or available longitudinal imaging are likely to differ systematically from those without such data, potentially reflecting differences in clinical severity, follow-up intensity, or care pathways. As a result, subgroup and longitudinal findings should be interpreted cautiously and should not be assumed to be representative of the entire cohort.

The interpretation of linear ventricular measurements must be considered in light of population-specific and demographic factors. In the present study, the thresholds of these measurements were not used to assert normative or pathological states, but rather as pragmatic reference values to explore measurement behavior and clinical alignment within a defined cohort. The development of population-adapted reference ranges and normative datasets remains an important goal for future hydrocephalus research.

### 4.1. Limitations

Although imaging biomarkers play an important role in the diagnosis and monitoring of hydrocephalus, the findings of this study must be interpreted within clear methodological and biological constraints. This was a single-center, retrospective study conducted at a Romanian tertiary-care hospital in a small, ethnically homogeneous cohort, with limited availability of individual-level socioeconomic data, restricting generalizability to other populations and healthcare contexts.

The cohort included a heterogeneous mix of hydrocephalus etiologies, encompassing both acute and chronic pathophysiological processes, for which ventricular enlargement does not carry uniform biological or clinical meaning. As such, imaging measurements should not be interpreted as etiologically interchangeable, nor as supporting uniform diagnostic thresholds or treatment expectations. Associations were not adjusted for potential confounders such as age, etiology, or comorbidity, as multivariable modeling was not feasible given the modest sample size and small subgroup counts.

The retrospective design and non-standardized imaging schedules resulted in substantial missing data and limited availability of paired cross-modality and longitudinal measurements, with unknown missingness mechanisms. Ventriculoperitoneal shunt placement reflects individualized clinical decision-making rather than an independent biological endpoint, precluding causal inference and introducing potential incorporation bias. Some patients underwent multiple CSF diversion procedures, but longitudinal analyses were not stratified by device sequence or number. Finally, variability in imaging technologies and acquisition parameters reflects real-world practice but increases measurement uncertainty and limits individual-level interpretation. Overall, these findings require validation in larger, multi-center cohorts with diverse etiologies and practice settings.

### 4.2. Implications and Future Directions

All findings should be interpreted within the context of a single-center study and reflect institutional imaging practices rather than universal standards of hydrocephalus assessment. To confirm these findings, future research should concentrate on prospective, multicenter studies that employ standardized imaging techniques and have predetermined follow-up periods. Combining ventricular measures with clinical and etiologic data can improve risk assessment as well as personalized therapy planning.

Although our cohort represented a variety of hydrocephalus etiologies, formal subtype-specific analyses were not possible due to small sample counts and insufficient modality pairing. As a result, our findings reflect the reliability of linear ventricular measures across imaging modalities, rather than etiology-specific behavior. While CT-MRI agreement appears to be consistent across clinical contexts, the interpretation of these measurements, particularly in chronic forms such as iNPH, must be guided by clinical presentation and additional imaging markers.

Recent advances in artificial intelligence and deep learning have enabled automated ventricular segmentation, volumetric analysis, and prognostic modeling in hydrocephalus, with several studies demonstrating promising diagnostic and predictive performance using imaging-based approaches. However, such performance metrics must be interpreted cautiously, as many AI-based approaches rely on relatively homogeneous, single-center, or highly selected datasets. Limited representativeness and inadequate external validation may result in degraded performance when models are applied to demographically, clinically, or institutionally diverse populations, with the potential to amplify existing health disparities.

However, the apparent ‘performance gap’ between automated volumetric methods and traditional linear measurements reflects not only differences in measurement precision but also substantial differences in data requirements, computational infrastructure, and clinical deployment. Automated pipelines typically rely on standardized MRI protocols, curated training datasets, and extensive external validation, which limits their immediate applicability in routine clinical settings where CT remains the primary modality and imaging protocols are heterogeneous.

In contrast, linear ventricular indices offer modality-agnostic, rapid, and reproducible measurements that can be applied across CT and MRI without specialized software. While these measures lack the granularity of full volumetric segmentation and are insufficient as standalone prognostic tools, they are still widely used. To ensure that digital technologies augment rather than replace clinical judgment in hydrocephalus care, such strategies will require rigorous validation, openness, and ethical oversight. Future research should also assess whether automated CT-based volumetric biomarkers, generated via deep-learning segmentation of ventricular CSF, can complement basic markers in everyday practice, particularly in locations where CT is the dominant modality and MRI is not possible [35].

Future studies should directly correlate the magnitude of change in linear ventricular measurements with standardized clinical outcome measures, such as the modified Rankin Scale or disease-specific grading systems for normal-pressure hydrocephalus. Establishing such links would help determine whether small imaging changes translate into meaningful functional improvement or clinical deterioration and would provide a clearer framework for evaluating both manual and automated measurement approaches.

## 5. Conclusions

In this single-center cohort, commonly used linear ventricular measurements constituted reliable and accessible methods for assessing adult hydrocephalus. Evans index and third-ventricle width showed high agreement between CT and MRI at the group level, indicating measurement comparability across modalities under routine institutional imaging conditions. Longitudinal analyses revealed modest but continuous reductions in ventricular size, with modest reductions observed following VP shunt placement; however, the clinical relevance of these changes remains uncertain. Higher Evans index values and the presence of transependymal CSF exudation were linked to increased ventriculoperitoneal shunt utilization, but no single imaging measure predicted treatment decisions. These findings support the role of linear imaging measurements as adjunctive tools that may inform, but should not replace, comprehensive clinical evaluation, and should be interpreted as evidence of measurement behavior across imaging modalities rather than as support for an etiologically uniform interpretation of ventricular enlargement or treatment response.

## Figures and Tables

**Table 1:** Baseline demographic, clinical, and imaging characteristics of the study cohort. Continuous variables are reported as median [interquartile range], and categorical variables as number (%). Imaging features were assessed on available examinations, resulting in variable denominators across variables (reported as *n/N* where applicable). Etiological categories reflect clinical diagnosis at presentation.

Characteristic	Value
**Patients, ** ***n***	68
**Sex—female, ** ***n*** **(%)**	36 (52.9%)
**Sex—male, ** ***n*** **(%)**	32 (47.1%)
**Age, years—median [IQR]**	61.0 [57.0–67.2]
**White/European, ** ***n*** **(%)**	68 (100%)
**Other race/ethnicity**	0
**Evans index—median [IQR]**	0.42 [0.39–0.49]
**Third-ventricle width (mm)—median [IQR]**	23.5 [18.2–26.0]
**Transependymal CSF exudation present, ** ***n*** **/** ***N*** **(% )**	30/52 (57.7%)
**Ventricular pattern—triventricular, ** ***n***	44
**Ventricular pattern—tetraventricular, ** ***n***	16
**Ventricular pattern—biventricular, ** ***n***	8
**Callosal angle, °**	
• Median [IQR]	82 [70–95]
• <80°	28 (41.2%)
• 80–100°	24 (35.3%)
• >100°	16 (23.5%)
**Temporal hornwidth, mm**	
• Median [IQR]	8.0 [6.0–11.0]
• <6 mm	24 (35.3%)
• ≥6 mm	44 (64.7%)

Baseline values represent the first available measurement for each parameter; measurements were not required to be concurrent across parameters.

**Table 2:** Device utilization summary within the study cohort. The table reports ventriculoperitoneal (VP) shunt and external ventricular drain (EVD) utilization among adult patients with hydrocephalus in a single-center tertiary-care cohort.

Metric	Value
VP shunt (ever), *n* (%)	26 (38.2)
EVD (ever), *n* (%)	22 (32.4)
Total device events (VP placements + EVD placements)	48
VP share of all device uses, %	54.2
EVD share of all device uses, %	45.8
VP − EVD difference, percentage points	5.8
VP:EVD count ratio	1.18
Devices per 10 patients	7.1
Devices per 100 patients	70.6

**Table 3:** Longitudinal changes in ventricular measurements based on complete-case follow-up data with heterogeneous intervals and imaging modalities.

Metric	Value
Patients with ≥2 dated scans, *n*	64
Inter-scan span (first → last), days—median [IQR]	33 [14–72]
ΔEvans (last–first)—*n*; median [IQR]	22; −0.02 [−0.06 to +0.01]
ΔV3 (last–first), mm—*n*; median [IQR]	16; −1.6 [−4.2 to +0.5]
Pre/Post VP pairs—Evans: *n*; median Δ [IQR]	10; −0.04 [−0.09 to −0.01]
Pre/Post VP pairs—V3: *n*; median Δ (mm) [IQR]	7; −3.0 [−6.5 to −0.8]

**Table 4:** CT–MRI agreement for linear ventricular measurements.

Metric	*n* Pairs	Correlation (r)	Mean Bias (MRI − CT)	95% Limits of Agreement	Median Absolute Difference
Evans index	41	0.93	−0.01	−0.07 to +0.05	0.02
Third-ventricle width (mm)	39	0.90	+0.6 mm	−3.2 to +4.4 mm	1.3 mm

**Table 5:** Association of baseline imaging markers with ever-VP. The table presents exploratory associations between baseline binary imaging features and subsequent VP shunt placement (“ever-VP”), expressed as odds ratios comparing shunted and non-shunted patients.

Marker (Baseline)	*n* with Marker Status	Odds Ratio (VP vs. No-VP)	*p*-Value
Transependymal CSF exudation present	38	3.33	0.26
Hemorrhage present	60	0.80	1
Mass effect	56	0.67	0.74
Basal cistern effaced	56	1.33	1

**Table 6:** Ventriculoperitoneal shunt utilization by Evans index strata in the overall cohort (etiologies combined for descriptive purposes; strata do not imply etiology-independent thresholds).

Evans Stratum	*n*	VP Rate (%)
0.30–0.39	4	0.0
0.40–0.49	12	50.0
≥0.50	4	100.0

**Table 7:** Diagnostic performance of rule-based imaging thresholds for VP shunt utilization.

Rule	Sens (%)	Spec (%)	PPV (%)	NPV (%)	Balanced Acc. (%)
Evans ≥ 0.40	66.7	65.0	57.1	73.7	65.8
Evans ≥ 0.40 + transependymal edema present	46.7	85.0	63.6	73.9	65.8
Evans ≥ 0.35	80.0	45.0	50.0	76.9	62.5
Transependymal edema present	33.3	96.7	83.3	73.7	65.0

## Data Availability

The original contributions presented in this study are included in the article. Further inquiries can be directed to the corresponding authors.

## References

[1] Anwar F., Zhang K., Sun C., Pang M., Zhou W., Li H., He R., Liu X., Ming D. (2024). Hydrocephalus: An Update on Latest Progress in Pathophysiological and Therapeutic Research. Biomed. Pharmacother..

[2] Milan J.B., Jensen T.S.R., Nørager N., Pedersen S.S.H., Riedel C.S., Toft N.M., Ammar A., Foroughi M., Grotenhuis A., Perera A. (2022). The ASPECT Hydrocephalus System: A Non-Hierarchical Descriptive System for Clinical Use. Acta Neurochir..

[3] Yakar H., Kuru Bektaşoğlu P., Gürer B. (2022). Clinical Diagnosis and Treatment Management of Normal Pressure Hydrocephalus. Cerebrospinal Fluid.

[4] Mirkhaef S.A., Harbaugh L., Nagra G. (2024). Hydrocephalus: A Review of Etiology-Driven Treatment Strategies. Cureus.

[5] Schwamb R., Dalpiaz A., Miao Y., Gonka J., Khan S.A. (2014). Clinical Manifestations of Hydrocephalus: A Review. Neurol. Clin. Neurosci..

[6] Roşu A.-I., Andrei D., Ghenciu L.A., Bolintineanu S.L. (2025). Hydrocephalus: Molecular and Neuroimaging Biomarkers in Diagnosis and Management. Biomedicines.

[7] Damasceno B.P. (2015). Neuroimaging in Normal Pressure Hydrocephalus. Dement. Neuropsychol..

[8] Zhang T., Zhou Y., Su G., Shi D., Gopinath S.C.B., Lakshmipriya T., Li S. (2019). Hydrocephaly Analysis Supported by Computerized Tomography and Nuclear Magnetic Resonance. J. Anal. Methods Chem..

[9] Pyrgelis E.-S., Velonakis G., Papageorgiou S.G., Stefanis L., Kapaki E., Constantinides V.C. (2023). Imaging Markers for Normal Pressure Hydrocephalus: An Overview. Biomedicines.

[10] Kwee R.M., Toxopeus R., Kwee T.C. (2024). Imaging Overuse in the Emergency Department: The View of Radiologists and Emergency Physicians. Eur. J. Radiol..

[11] Lassere M.N. (2008). Imaging: The Need for Standardization. Best Pract. Res. Clin. Rheumatol..

[12] Miskin N., Patel H., Franceschi A.M., Ades-Aron B., Le A., Damadian B.E., Stanton C., Serulle Y., Golomb J., Gonen O. (2017). Diagnosis of Normal-Pressure Hydrocephalus: Use of Traditional Measures in the Era of Volumetric MR Imaging. Radiology.

[13] Fleuren K.J.R., Koehler P.J., Hoff E.I. (2025). The History of the Evans Index. Eur. Neurol..

[14] Zhou X., Xia J. (2022). Application of Evans Index in Normal Pressure Hydrocephalus Patients: A Mini Review. Front. Aging Neurosci..

[15] Missori P., Paolini S., Peschillo S., Mancarella C., Scafa A.K., Rastelli E., Martini S., Fattapposta F., Currà A. (2022). Temporal Horn Enlargements Predict Secondary Hydrocephalus Diagnosis Earlier than Evans’ Index. Tomography.

[16] Kadaba Sridhar S., Dysterheft Robb J., Gupta R., Cheong S., Kuang R., Samadani U. (2024). Structural Neuroimaging Markers of Normal Pressure Hydrocephalus versus Alzheimer’s Dementia and Parkinson’s Disease, and Hydrocephalus versus Atrophy in Chronic TBI—A Narrative Review. Front. Neurol..

[17] Crook J.E., Gunter J.L., Ball C.T., Jones D.T., Graff-Radford J., Knopman D.S., Boeve B.F., Petersen R.C., Jack C.R., Graff-Radford N.R. (2020). Linear vs Volume Measures of Ventricle Size: Relation to Present and Future Gait and Cognition. Neurology.

[18] Reinard K., Basheer A., Phillips S., Snyder A., Agarwal A., Jafari-Khouzani K., Soltanian-Zadeh H., Schultz L., Aho T., Schwalb J. (2015). Simple and Reproducible Linear Measurements to Determine Ventricular Enlargement in Adults. Surg. Neurol. Int..

[19] Ebel F., Mariani C., Guzman R., Soleman J. (2025). Longitudinal Changes in Ventricular Volume after Treating Aqueduct Stenosis through Endoscopic Third Ventriculostomy in Adults. Fluids Barriers CNS.

[20] Ryska P., Slezak O., Eklund A., Salzer J., Malm J., Zizka J. (2021). Variability of Normal Pressure Hydrocephalus Imaging Biomarkers with Respect to Section Plane Angulation: How Wrong a Radiologist Can Be?. Am. J. Neuroradiol..

[21] Takagi K., Watahiki R., Machida T., Onouchi K., Kato K., Oshima M. (2020). Reliability and Interobserver Variability of Evans’ Index and Disproportionately Enlarged Subarachnoid Space Hydrocephalus as Diagnostic Criteria for Idiopathic Normal Pressure Hydrocephalus. Asian J. Neurosurg..

[22] Keong N.C.H., Pena A., Price S.J., Czosnyka M., Czosnyka Z., Pickard J.D. (2016). Imaging Normal Pressure Hydrocephalus: Theories, Techniques, and Challenges. Neurosurg. Focus.

[23] Kockum K., Lilja-Lund O., Larsson E.-M., Rosell M., Söderström L., Virhammar J., Laurell K. (2018). The Idiopathic Normal-Pressure Hydrocephalus Radscale: A Radiological Scale for Structured Evaluation. Eur. J. Neurol..

[24] Toma A.K., Holl E., Kitchen N.D., Watkins L.D. (2011). Evans’ Index Revisited: The Need for an Alternative in Normal Pressure Hydrocephalus. Neurosurgery.

[25] Mercado-Diaz L.R., Prakash N., Gong G.X., Posada-Quintero H.F. (2025). Artificial Intelligence Approaches for the Detection of Normal Pressure Hydrocephalus: A Systematic Review. Appl. Sci..

[26] Duan W., Zhang J., Zhang L., Lin Z., Chen Y., Hao X., Wang Y., Zhang H. (2020). Evaluation of an Artificial Intelligent Hydrocephalus Diagnosis Model Based on Transfer Learning. Medicine.

[27] Kordnoori S., Sabeti M., Goorakani Y., Tavassoli A., Moradi E. (2025). Artificial Intelligence-Based Discrimination of Hydrocephaly in Brain CT Scan. Comput. Methods Biomech. Biomed. Eng. Imaging Vis..

[28] Esteva A., Robicquet A., Ramsundar B., Kuleshov V., DePristo M., Chou K., Cui C., Corrado G., Thrun S., Dean J. (2019). A Guide to Deep Learning in Healthcare. Nat. Med..

[29] Topol E.J. (2019). High-Performance Medicine: The Convergence of Human and Artificial Intelligence. Nat. Med..

[30] Kelly C.J., Karthikesalingam A., Suleyman M., Corrado G., King D. (2019). Key Challenges for Delivering Clinical Impact with Artificial Intelligence. BMC Med..

[31] Yadav R.P., Yadav S.N. (2025). Measurement of Brain Ventricles Using Multidetector Computed Tomography: A Prospective Study at TU Teaching Hospital, Maharajgunj, Nepal. Nepal Med. J..

[32] Virhammar J., Laurell K., Cesarini K.G., Larsson E.M. (2014). The callosal angle measured on MRI as a predictor of outcome in idiopathic normal-pressure hydrocephalus. J. Neurosurg..

[33] Muthalagan D., Tan K.H., Sapiai N.A., Mohd Ismail Z.I., Mukhtar S.F., Ng W.S., Mah S.C. (2025). Delineating Neuroanatomical Structures for the Measurement of Temporal Horn Dilatation. Malays. J. Med. Sci..

[34] Yang P.H., Almgren-Bell A., Gu H., Dowling A.V., Pugazenthi S., Mackey K., Dupépé E.B., Strahle J.M. (2022). Etiology- and region-specific characteristics of transependymal cerebrospinal fluid flow. J. Neurosurg. Pediatr..

[35] Srikrishna M., Seo W., Zettergren A., Kern S., Cantré D., Gessler F., Sotoudeh H., Seidlitz J., Bernstock J.D., Wahlund L.-O. (2024). Assessing CT-Based Volumetric Analysis via Transfer Learning with MRI and Manual Labels for Idiopathic Normal Pressure Hydrocephalus. medRxiv.

[36] Srikrishna M., Ashton N.J., Moscoso A., Pereira J.B., Heckemann R.A., Van Westen D., Volpe G., Simrén J., Zettergren A., Kern S. (2024). CT-Based Volumetric Measures Obtained through Deep Learning: Association with Biomarkers of Neurodegeneration. Alzheimer’s Dement..

[37] Wen J., Liang X., Zeng L., Xiao Q., Zhang L., Yang X., Zeng Y., Wu Z., Zhang Y., Su Z. (2025). Accuracy and Clinical Application Prospects of Mobile Low-Field Strength Magnetic Resonance Imaging in the Diagnosis of Hydrocephalus: A Retrospective Cohort Study. Brain Res. Bull..

[38] Kockum K., Virhammar J., Riklund K., Söderström L., Larsson E.-M., Laurell K. (2019). Standardized Image Evaluation in Patients with Idiopathic Normal Pressure Hydrocephalus: Consistency and Reproducibility. Neuroradiology.

[39] Cogswell P.M., Murphy M.C., Senjem M.L., Botha H., Gunter J.L., Elder B.D., Graff-Radford J., Jones D.T., Cutsforth-Gregory J.K., Schwarz C.G. (2021). Changes in Ventricular and Cortical Volumes Following Shunt Placement in Patients with Idiopathic Normal Pressure Hydrocephalus. Am. J. Neuroradiol..

[40] Bradley W.G., Scalzo D., Queralt J., Nitz W.N., Atkinson D.J., Wong P. (1996). Normal-Pressure Hydrocephalus: Evaluation with Cerebrospinal Fluid Flow Measurements at MR Imaging. Radiology.

[41] Yamada S., Ishikawa M., Yamaguchi M., Yamamoto K. (2019). Longitudinal Morphological Changes during Recovery from Brain Deformation Due to Idiopathic Normal Pressure Hydrocephalus after Ventriculoperitoneal Shunt Surgery. Sci. Rep..

[42] Ruprecht R., Scheurer E., Lenz C. (2019). Systematic Review on the Characterization of Chronic Traumatic Encephalopathy by MRI and MRS. J. Magn. Reson. Imaging.

[43] Relkin N., Marmarou A., Klinge P., Bergsneider M., Black P.M.L. (2005). Diagnosing Idiopathic Normal-Pressure Hydrocephalus. Neurosurgery.

[44] Thavarajasingam S.G., El-Khatib M., Vemulapalli K., Iradukunda H.A.S., Vishnu S.K., Borchert R., Russo S., Eide P.K. (2022). Radiological Predictors of Shunt Response in the Diagnosis and Treatment of Idiopathic Normal Pressure Hydrocephalus: A Systematic Review and Meta-Analysis. Acta Neurochir..

[45] Luikku A.J., Hall A., Nerg O., Koivisto A.M., Hiltunen M., Helisalmi S., Herukka S.-K., Sutela A., Kojoukhova M., Mattila J. (2016). Multimodal Analysis to Predict Shunt Surgery Outcome of 284 Patients with Suspected Idiopathic Normal Pressure Hydrocephalus. Acta Neurochir..

